# Chronic Experimental Diabetes Accelerates Urinary Elimination of Deprenyl and its Metabolites

**DOI:** 10.2174/1874104500802010001

**Published:** 2008-01-10

**Authors:** Ernest Adeghate, Péter Sótonyi Jr, Huba Kalász

**Affiliations:** 1 Department of Anatomy, Faculty of Medicine & Health Sciences, United Arab Emirates University, Al Ain, P.O. Box 17666, United Arab Emirates; 2 Department of Cardiovascular Surgery, Semmelwies University, Budapest, Hungary; 3 Department of Pharmacology and Pharmacotherapy, Semmelweis University, Budapest, P.O. Box 370, Hungary 1445

**Keywords:** Deprenyl, diabetes, pharmacokinetics, urinary elimination.

## Abstract

Many diabetic patients take several medications to treat diabetes-associated complications and other ailments. The mode of elimination of these drugs and their metabolites are poorly understood. The elimination of deprenyl, a MAO-B inhibitor, used for the treatment of the early stage of Parkinson’s disease and senile dementia was investigated using thin layer chromatography.

Male Wistar rats (180-200 g) were rendered diabetic by streptozotocin (STZ) treatment (60 mg/kg, i.v.). Rats having at least three times higher plasma glucose level than the normal were considered diabetic. Rats were treated with a single oral dose of 5 mg/kg ^14^C-(methyl)-labeled (-)-deprenyl, 98 µCi/mg. Diabetic rats excreted the majority of urinary radioactivity in 8 hours, while control rats did it in 16 hours. The approximate ratio of major metabolites as determined using thin-layer chromatography did not change. In conclusion, diabetic rats excreted radiolabelled-deprenyl more rapidly compared to control animals. Increased elimination of deprenyl should be taken into account in the management of patients suffering from diabetes.

## INTRODUCTION

The route and mode of elimination of some drugs such as paracetamol has been studied in the saliva of type 1 and type 2 diabetic patients [1] and diflunisal in animal model of diabetes [2]. In most of the reports on drug elimination, the controversial issues are the kinetics, route of elimination, metabolite formation and the mechanism by which they are eliminated.

Oxidative N-demethylation is one of the basic metabolic reactions of both xenobiotics and endogenous compounds of vertebrates [3-5]. Removal of the methyl group decreases the lipophilicity of the compounds, thereby facilitating their excretion. A classical model of metabolic demethylation is methylamine which is generated endogenously [5]. The sources of methylamine include adrenaline, creatinine and sarcosine [5].

Metabolic studies on drugs and food components have dealt mainly with the identification and quantitative determination of the N-demethylated compounds [3-6]. Much less, if any attention has been focused on the fate of the molecular fragment derived from the methyl group. However, it is well known that formaldehyde is produced by N-demethylation and has been widely cited by many textbooks of pharmacology. The generated formaldehyde was easily identified when the N-methyl group was ^14^C radiolabelled; and certain N-demethylation reactions occurred through the homocystein-methionine cycle resulting in methylation of an endogenous compound [5].

Recent publications stated that formaldehyde, formed *in situ,* can directly take part in methylation reactions [5, 7-9]. Tyihák E, *et al*. [7] and Trezli *et al*. [8] reported a spontaneous methylation of N-amino group of lysine by formaldehyde. The application of planar chromatography, especially two-dimensional thin-layer chromatography has given an easy and reliable tool to locate the formaldehyde after pre-chromatography reaction with dimedone [10-13]. Although, formaldehyde was eliminated in urine, further reaction also took place in which a basic amino acid (lysine) is the major target. The methylation of lysine results in N^ε^-monomethyllysine, which was identified using thin-layer chromatography and high-performance liquid chromatography combined with mass spectrometry detection (HPLC-MS) [14-17]. Direct verification of the N-methyl transfer from the drug [(-)-deprenyl] to lysine was also done using ^14^C-methyl labeled deprenyl, and one part of the radioactivity was detected on the N-monomethyllysine.

Studies by Yu *et al*. [18,19] detailed the significance of semicarbazide-sensitive amine oxidase (SSAO) in the generation of formaldehyde from exogenous and endogenous compounds as well as the level of SSAO in Type 2 diabetes mellitus. Other investigators reported the association of serum monoamine oxidase level and retinopathy in diabetes mellitus [20].

The present report examined the effect of diabetes mellitus on the urinary excretion of radiolabelled deprenyl [(R)-(-)-^14^C-N-methyl-2-methyl-N-propynyl-phenylethylamine hydrochloride].

## MATERIALS AND METHODS

### Solvents and Chemicals

All solvents were of HPLC grade of purity, purchased from E. Merck (Darmstadt, Germany). HPLC grade water was used.

### Methods

#### Treatments

Male Wistar rats (180-200 g) were treated *per os *with 5 mg/kg radiolabelled L-deprenyl. Urine was collected at 2, 4, 6, 8, 16 and 24 hours after the administration of radiolabelled L-deprenyl.

Male Wistar rats (180-200 g) were rendered diabetic by streptozotocin (STZ) treatment (60 mg/kg, i.v.). Blood glucose level and body weight were monitored weekly. Rats having at least three times higher plasma glucose than the normal ones were considered diabetic. Control rats did not receive STZ. Both diabetic and control rats were administered with a single oral dose of 5 mg/kg ^14^C-(methyl)-labeled (-)-deprenyl, 98 µCi/mg. Drinking water supply to rats was *ad libitum*. Animal treatment was conducted under protocols in accordance with the Guide for Care and Use of Laboratory Animals (Semmelweis University, Budapest, Hungary). After the protocol was supervised by the Ethical Committee of Animal Research, the permission was granted by Budapest Animal Health and Food Control Station, Budapest, Hungary (No. 1810/003/2004),

Metabolites were identified and quantified using thin-layer chromatography (TLC) on TLC silica plates F_254_, 20 x 20 cm (E. Merck), developed with chloroform—methanol—water (7:5:1, 1^st^ dimension) and (triethanolamine—chloroform (5:95, 2^nd^ dimension, displacement development) mobile phases. The spots on the TLC plates were evaluated using digital autoradiography (DAR of Berthold, Germany).

#### Pre-Column Derivatization with Fmoc Chloride

To every 2 mL urine sample, 1 mL potassium borate buffer (0.8 M, pH 10) was added, and the mixture was vigorously shaken for 1 min using a mixer. Fmoc chloride solution in acetonitrile (2 mL, 10 mM) was then added, and vortexed immediately for 1 min. Subsequently 2 mL n-hexane was added, the mixture shaken for 1 min, and the upper and lower phases were separated by centrifugation. The upper (organic) phase containing the excess of Fmoc reagent was discarded. The n-hexane extraction was repeated twice. Finally, 200 µL of acetic acid (10%, v/v) was added to the samples, mixed, and 100 µL sample was subjected to HPLC separation. The solution of standard N^ε^-Methyllysine HCl was also carried out through an identical derivatization process.

#### HPLC

A JASCO system (JASCO Corporation, Tokyo, Japan) consisting of a DG-208054 degasser, PU-1580 pump, AS-2057 plus automatic sample injector, and UV-1575 detector at 265 nm were used. Chromatographic data were stored and evaluated using the SRI Model 202 Peak Simple Chromatographic Data System (SRI Instruments, Torrance, CA, USA). The standard N^ε^-Monomethyl- lysine HCl and urine samples were separated after derivatization with Fmoc hydrochloride. HPLC was carried out using a 25 cm x 4.6 mm I.D. column packed with 6 µm Kovasil C18 endcapped particles (Chemie Uetikon, Uetikon, Switzerland) to separate the radioactive (^14^C-labelled monomethyllysine) peak of the urine using acetonitrile—water (2:1), which also contain 0.1% formic acid as the mobile phase. The HPLC column was kept at 28 ºC. The mobile phase flow rate was 1.5 mL min^-1^, and each fraction was collected for 1 min.

## RESULTS

**Fig. (1):**
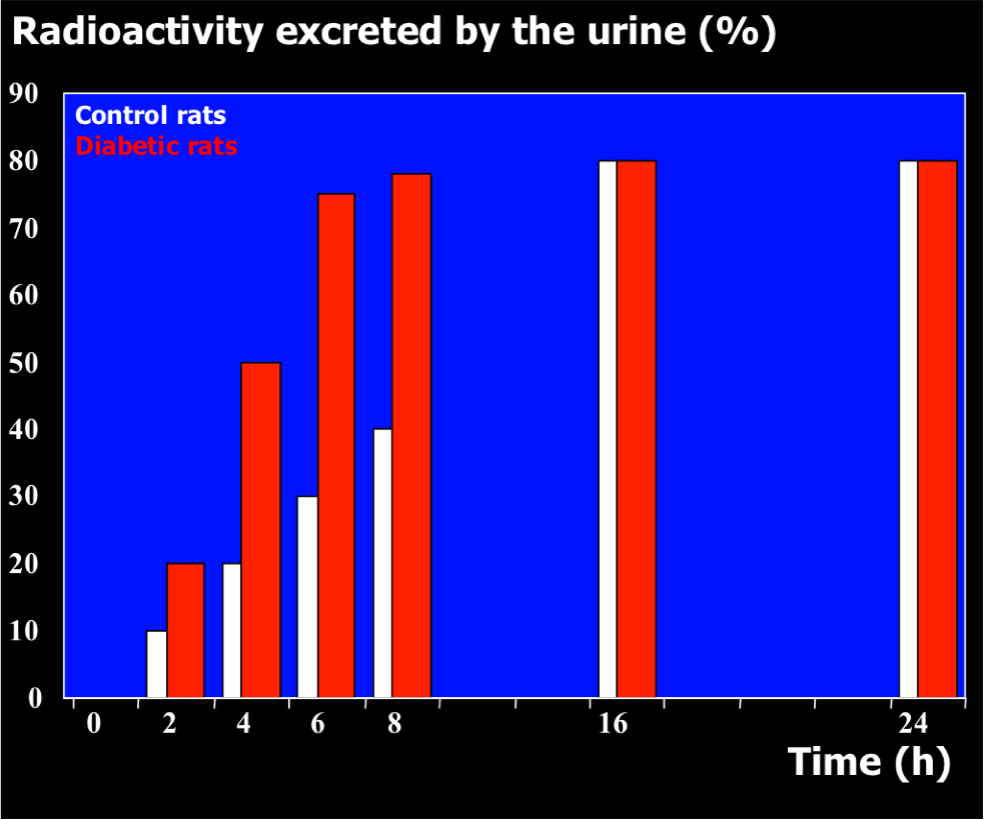
The time course of urinary excretion of radioactivity when the N-methyl group of (-)-deprenyl was ^14^C-labelled.

**Fig. (2):**
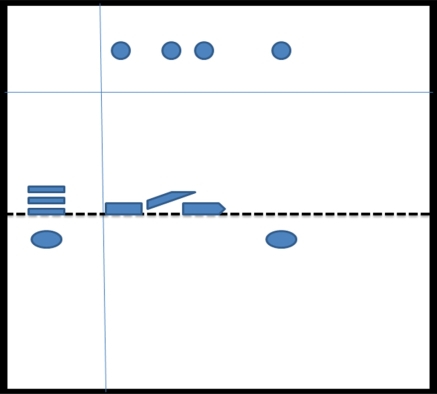
Thin-layer chromatography of radiolabelled deprenyl metabolites.

Rat urine was collected following oral administration of ^14^C-(methyl)-labeled (-)-deprenyl, and the cumulative excretion of radioactivity was determined. Urinary elimination of radiolabelled-deprenyl was significantly (p < 0.05) faster in the first 8 hours after oral administration of ^14^C-(methyl)-labeled (-)-deprenyl, when compared to controls (Fig. **1**). STZ-induced diabetes facilitated urinary elimination of the radioactivity. The rat urine (containing radiolabelled-deprenyl and its metabolites) was subjected to two-dimensional thin-layer chromatography after reacting it with dimedon, and radioactive spots were located using an X-ray film (Fig. **2**). The spots were scarped, and their ratios were compared. Fig. (**3**) showed the major metabolites of deprenyl. The ratio of methamphetamine, p-hydroxy-metham-phetamine, deprenyl and formaldehyde were 20:3:1:1 in the urine collected for 8 hours (Table **1**). Urine eliminated radioactivity in N-monomethyllysine was determined using HPLC, its amount was about 10% of the free urinary formaldehyde.

## DISCUSSION

There are two major metabolites of (-)-deprenyl. The detection of these metabolites depends on the analytical method applied. Reynolds *et al*. [21] detected methamphetamine about 30 years ago, as the major deprenyl metabolite in man. They used GC-MS (gas chromatography combined with mass spectrometry), so their method did not make it possible to find the other major deprenyl metabolite, deprenyl-N-oxide that was later identified by Katagi *et al*. [22]. Unfortunately, deprenyl-N-oxide cannot be detected using thin-layer chromatography either. Other deprenyl metabolites include para-hydroxy-methamphetamine, nordeprenyl (also called desmethyldeprenyl), amphetamine, and phenylacetone. However, the radiolabelled N-methyl group is present only in para-hydroxy-methamphetamine. Moreover, Szatmári described 25 deprenyl metabolites with aromatic ring, as well as two more fragments containing the propargyl (N-methyl-propargylamine and propargylamine) as well as propiolaldehyde [23]. If we also calculate the conjugates of several deprenyl metabolites (mainly that of p-hydroxy-metabolites), the gross number of metabolic products can approach 40.

It is peculiar that previous studies on deprenyl metabolism neglected an evident metabolite of deprenyl, the formaldehyde. In fact, most of the basic textbooks on pharmacology show that formaldehyde is a byproduct of R-X-CH_3_ splitting, where the R-X-CH_3_ R-X-H + CH_2_O reaction can be either N-demethylation, or O-demethylation or S-demethylation [3, 4, 6].

Our earlier papers have shown that ^14^C-labelled deprenyl is generated from ^14^C-labelled (-)-deprenyl which is eliminated in urine. Moreover, one of the radiolabelled peaks of the same rat urine was ^14^C-labelled-N^ε^-monomethyl- lysine. Identification of N^ε^-monomethyllysine in the urine as well as in isolated homogenous peak with radioactivity was done by the use of two different HPLC-MS techniques that used electrospray (ES) ionization [24] as well as atmospheric pressure chemical ionization (APCI) modes [17]. It is also worth noting that the generated compounds were present in the urine only in traces (formaldehyde, about 1 to 2% of radioactivity administered, and N^ε^-monomethyllysine, about 0.1%).

The responsible enzyme of oxidative demethylation (or one of the responsible enzymes) is semicarbazide-sensitive amine oxidase (SSAO; EC.1.4.3.6), which shows an elevated level in the blood of diabetic patients [25, 26].

In contrast, hepatic cytochrome P450 activity of aminopyrine N-demethylase and ethoxyresorufin O-deethylase enzymes showed changes in opposite directions after the onset of STZ-induced diabetes. Aminopyrine N-demethylase activity significantly decreased [2.88±0.16 (control), 2.19±0.46 (after treatment with 50 mg/kg STZ, p≤0.001) and 1.53±0.22 (after treatment with 70 mg/kg STZ, p≤0.001)], while ethoxyresorufin O-deethylase activity increased [49.6±5.0 (control), 73.3±12.6 (after treatment with 50 mg/kg STZ, p≤0.01) and 82.6±14.9 (after treatment with 70 mg/kg STZ, p≤0.01)].

Watanabe *et al*. [27] reported that the change in pharmacokinetics after STZ-induced diabetes t_max_ became shorter, however, C_max_ and the area under the time versus concentration curve (AUC) became higher in the case of diabetic rats compared to control rats. Watanabe *et al*. [27] also compared the parameters of pharmacokinetics in the spontaneously diabetic (GK-rats, NIDDM model) and Zucker rats (almost normal glycemic but abnormally glucose tolerant, hyperinsulinemic, increasingly obese, insulin-resistant rats). These rats showed even shorter t_½_, but higher C_max_ and AUC parameters.

## CONCLUSION

Urinary elimination of (-)-deprenyl and its metabolites was faster in STZ-induced diabetic rats compared to control. As the metabolites and their ration was not changed, more frequent treatment with lower dose, or the use of sustained release preparations can counterbalance the faster elimination of deprenyl and possibly other drugs used in diabetes.

## Figures and Tables

**Fig. (3):**
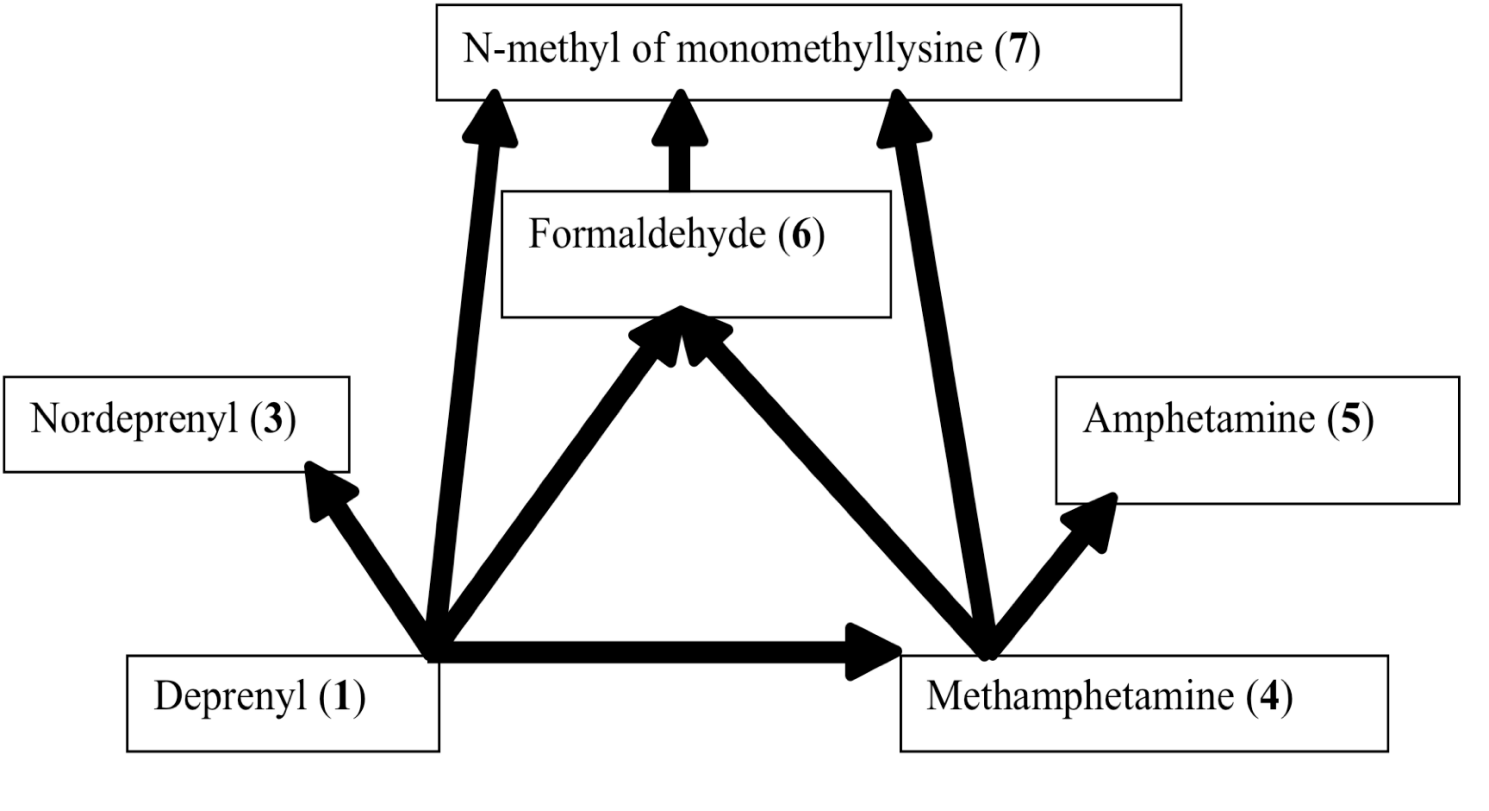
Major metabolic routes and metabolites of (-)-deprenyl. (The number in bracket corresponds to those in Table **1**)

**Table 1:** The Name, Chemical Structure, Presence of Radioactivity and logP Value of Deprenyl and its Major Metabolites

	Compound	Structure	Radioactivity	logP
**1**	Deprenyl	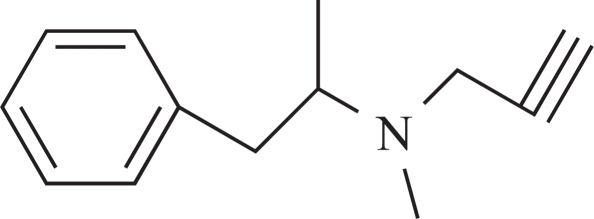	_+_	3.31
**2**	Deprenyl-N-oxide	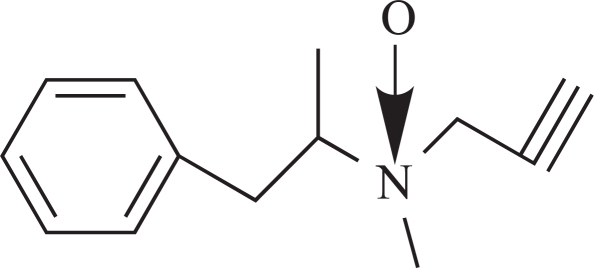	+	2.77
**3**	Nordeprenyl	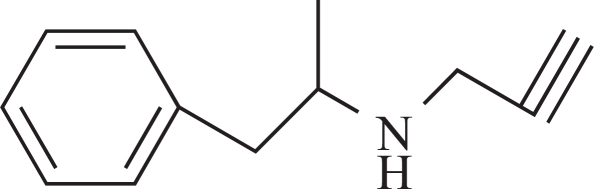	-	2.66
**4**	Methamphetamine	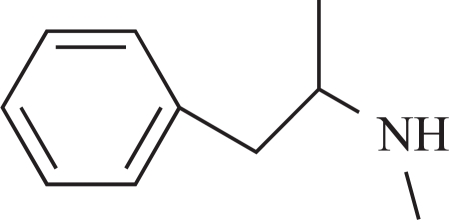	+	2.03
**5**	Amphetamine	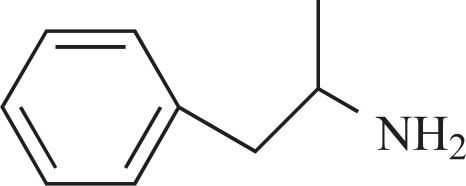	-	1.57
**6**	Formaldehyde	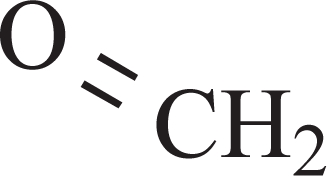	+	0.07
**7**	Monomethyllysine	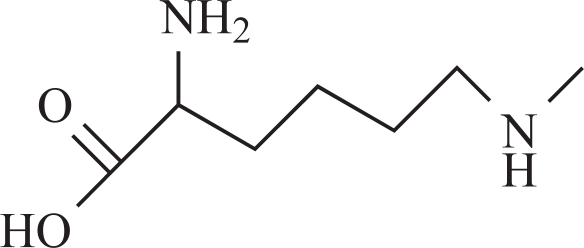	+	-2.36
**8**	Lysine	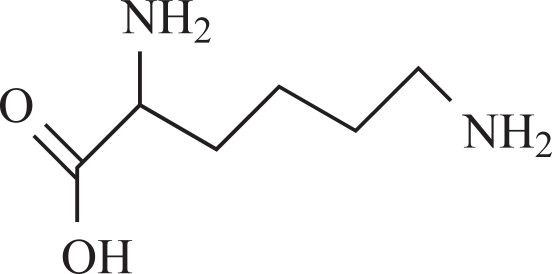	-	-2.92
